# Repurposing drugs that slow down aging for the prevention of Alzheimer’s disease

**DOI:** 10.1002/alz.089109

**Published:** 2025-01-03

**Authors:** Simone Tambaro, Andrea Mosqueda Solis, Bowen Tang, Thais Lopes De Oliveira, Chenxi Qin, Alan Kavsek, Arvid Sjölander, Kristina Johnell, Dorota Religa, Christian Riedel, Per Nilsson, Sara Hägg

**Affiliations:** ^1^ Karolinska Institutet, Solna Sweden; ^2^ Karolinska Institutet, Huddinge Sweden; ^3^ Division of Clinical Geriatrics, Center for Alzheimer Research, Department of Neurobiology, Care Sciences and Society, Karolinska Institutet, Stockholm Sweden

## Abstract

**Background:**

High age is the biggest risk factor for Alzheimer’s disease (AD). Approved drugs that slow down the aging process have the potential to be repurposed for the primary prevention of AD. The aim of our project was to use a reverse translational approach to identify such drug candidates in epidemiological data followed by validation in cell‐based models and animal models of aging and AD.

**Method:**

Longitudinal samples from a Swedish twin cohort (N = 672) were used to assess the effects of commonly prescribed medications on biological aging. Mendelian Randomization was used to identify associations between genetic variations in the glucagon‐like peptide 1 (*GLP‐1*) analogues target and the risk of developing AD. The identified classes of compounds were next evaluated in a pipe line of cellular and animal models for their potency of increasing lifespan using *C. elegans* models expressing amyloidbeta (Aβ) and tau, and for lowering Aβ levels in neuroblastoma SH‐SY5Y cells and AD‐associated pathologies in *App^NL‐F^* knock‐in mice as well as their anti‐aging effects in a novel mouse model of CNS aging.

**Result:**

We found that initiation of calcium channel blockers (CCBs) was associated with about a two‐year decrease in multiple epigenetic clocks (DNA methylation ages) as well as in functional biological ages. Moreover, genetic variants in GLP‐1 targets were associated with lower risk of developing AD. Several of the clinically used CCBs significantly increased the life span in *C. elegans* Aβ model and decreased levels of secreted Aβ from SH‐SY5Y cells. Several GLP‐1 analogues, similar to the CCBs, demonstrated a positive effect in reducing Aβ42 levels in SH‐SY5Y cell media.

**Conclusion:**

These preliminary data, demonstrating an extension of lifespan and a reduction in Aβ42 production, support the potential repurposing strategy for CCBs and GLP‐1 analogs in slowing down aging and preventing/treating AD. The investigation of these compounds in mouse models of aging and AD will provide a more complete picture of the therapeutic potential and may eventually identify novel preventive measures for AD within CCB and GLP‐1 analogs.

